# Functional Shifts in Gut Microbiota and Associated Metabolites Suggest Gut–Brain Axis Dysregulation in Pediatric Autoimmune Neuropsychiatric Disorders Associated with Streptococcal Infections (PANDAS)

**DOI:** 10.3390/microorganisms14051036

**Published:** 2026-05-02

**Authors:** Shabana M. Shaik, Gabriele Schiro, Daniel Laubitz, Juliette C. Madan, Connor P. Kelley, Michael Daines, Sydney A. Rice, Fayez K. Ghishan, Pawel R. Kiela

**Affiliations:** 1 Department of Pediatrics, Daniel Cracchiolo Institute for Pediatric Autoimmune Disease Research, Steele Children’s Research Center, University of Arizona, Tucson, AZ 85721, USA; shabanashaik@arizona.edu (S.M.S.); laubitz@arizona.edu (D.L.);; 2 Neuroimmune Psychiatric Disorders Program, Division of Child Psychiatry, Departments of Psychiatry & Pediatrics, Dartmouth Hitchcock Medical Center, Lebanon, NH 03756, USA; 3 Department of Epidemiology, Geisel School of Medicine at Dartmouth, Hanover, NH 03756, USA; 4 Children’s Postinfectious Autoimmune Encephalopathy (CPAE) Center of Excellence, Steele Children’s Research Center, Department of Pediatrics, University of Arizona, Tucson, AZ 85724, USA; 5 Department of Immunobiology, University of Arizona, Tucson, AZ 85721, USA

**Keywords:** neuropsychiatric, microbiome, metabolome, children, nose, throat, fecal, 16S, metagenomics

## Abstract

**Background:** Pediatric Autoimmune Neuropsychiatric Disorders Associated with Streptococcal infections (PANDAS) are characterized by neuropsychiatric symptoms linked to immune dysregulation. Emerging evidence highlights the role of host–microbiome interactions in modulating neuro-immune functions via gut–brain axis signaling; however, its contribution to PANDAS pathophysiology remains poorly understood. **Methods:** We conducted microbiome analysis from samples collected across multiple sites of PANDAS patients including nasal, throat and stool. We performed an integrated multi-omics analysis of stool samples from pediatric PANDAS cases and healthy controls, including discordant twin pairs. Microbial composition and function were assessed using 16S rRNA gene sequencing, shotgun metagenomics, while untargeted metabolomic profiling was performed using ultra-performance liquid chromatography-mass spectrometry (UPLC-MS/MS). **Results:** PANDAS cases exhibited reduced alpha diversity and significantly altered beta diversity compared to controls, indicating shifts in gut microbial composition. Shotgun metagenomic analysis revealed differential enrichment of functional pathways, including diminished quorum sensing, altered gamma-aminobutyric acid (GABA) biosynthesis, and microbial degradation processes. Multiple gut–brain modules (GBMs) and gut metabolic modules (GMMs) associated with neurotransmission, transport activities and metabolism were significantly perturbed in PANDAS. Metabolomic profiling showed reduced functional diversity and distinct clustering of metabolic profiles, with differential abundance of amino acids, bile acids, and neuroactive compounds. Integrative analysis further identified disrupted microbe–metabolite networks allied to gut–brain signaling. **Conclusions:** Our findings reveal significant functional shifts in gut microbiota composition, functional capacity and metabolite profile in PANDAS, suggesting dysregulation of the gut–brain axis signaling. This study provides a foundation for development of microbiome-based biomarkers and therapeutic strategies for pediatric neuropsychiatric disorders.

## 1. Introduction

Autoimmune encephalopathy (AE) is a neurological disorder caused by autoantibodies that target the neurons in the brain to elicit inflammatory immune responses [1,2]. While AE can occur at all ages, Children’s Post-Infectious Autoimmune Encephalopathy (CPAE) also known as the Pediatric Acute Neuropsychiatric Syndrome (PANS) [3,4,5] is an increasingly recognized neuropsychiatric disorder. Pediatric Autoimmune Neuropsychiatric Disorders Associated with Streptococcal infection (PANDAS), originally described by Swedo et al. [6], is considered to be a subset of PANS and is defined by the temporal association with Group A beta-hemolytic streptococcal infection, prepubertal acute onset, obsessive–compulsive disorder (OCD) and/or tic disorder, episodic symptom course, and accompanying neurological and behavioral abnormalities. PANDAS is a rare condition, with a retrospective study across three U.S. academic centers estimating its annual incidence at approximately 1 in 11,765 children aged 3–12 years [7].

The pathophysiology of PANDAS remains incompletely understood, although there is increasing evidence that immune dysregulation plays a key role [8,9,10,11] and the presence of autoantibodies capable of recognizing a variety of antigens, including cholinergic interneurons (CINs) in the striatum [12,13], strongly suggests an autoimmune background and systemic inflammation. Despite these advances, several critical knowledge gaps remain. Specifically, there is (i) a lack of causal evidence linking microbial dysbiosis to disease onset or exacerbation, (ii) limited investigation of microbiota beyond the gut, particularly at mucosal sites relevant to infection, and (iii) insufficient mechanistic understanding of how host–microbe interactions influence immune and neurodevelopmental signaling pathways in PANDAS.

The gut microbiome has emerged as a key component of a bidirectional communication path between the gut and the brain [14]. A growing body of data from animal models, significantly from those raised in germ-free conditions shows that intestinal microbes play a crucial role in the host brain development [15] and remain involved in regulating neurobehavior throughout life [16]. Recent studies further demonstrate that microbiota–gut–brain communication is mediated through integrated immune, neural and metabolic pathways, including cytokine signaling, microglial activation, and neurodevelopmental processes [17]. Microglia, infiltrating myeloid cells, astrocytes, oligodendrocytes along with blood–brain barrier (BBB) and cytokine signaling, are all critical components of the central nervous system (CNS) [18]. Gut microbes produce diverse metabolic products that can stimulate the host immune system, modulate inflammatory responses, and support immune homeostasis. The signaling molecules from the gut can reach the CNS via the lymphatic and systemic circulation or via direct interactions with the vagus nerve to modulate brain function [19]. Microbial products such as short-chain fatty acids (SCFAs), biogenic amines, and amino acid (AA) derived metabolites can function as neurotransmitters or neuromodulators while lipopolysaccharide (LPS) or neurotoxic metabolites from pathogenic bacteria can mediate the synthesis of pro-inflammatory cytokines and activate a host’s immune response [20,21]. Importantly, gut microbes are shown to control maturation and function of microglia, the brain-resident immune cells that are critical for maintaining brain homeostasis [22]. They respond to changes in gut microbiome in a sex- and time-dependent manner [23] and can be activated by microbial-associated molecular patterns (MAMPs) to release reactive molecules that damage brain epithelial cells, and compromise the integrity of the BBB [24].

Importantly, microbiome–host interactions relevant to PANDAS may not be restricted to gut. The nasal and oral microbiota represent primary reservoirs for Group A *Streptococcus*, the key infectious trigger of PANDAS, and play a central role in mucosal immune priming. Emerging evidence suggests that microbial communities across body sites are interconnected, e.g., oral–gut axis, oral–brain axis, and that cross-site microbial interactions can influence systemic inflammation and blood–brain barrier integrity. Therefore, a multisite microbiome approach is biologically meaningful, as it captures both the sites of pathogen exposure and downstream systemic immune and neuropsychiatric effects.

Changes in the diversity and composition of the gut microbiota have been reported for a wide range of neurological diseases and neuropsychiatric disorders including autism spectrum disorder (ASD) [25], schizophrenia [26], and major depression disorders (MDD) [27,28]. A recent systematic review to analyze the microbiome involved in various neuropsychiatric diseases representing approximately 2100 cases and 2800 healthy controls provided new insights into gut and brain signaling via hypothalamic–pituitary–adrenal (HPA) axis [29]. Recent evidence further highlights the role of microbiome-driven inflammation and immune signaling in pediatric neurodevelopmental and neuropsychiatric disorders, supporting a unifying framework in which microbial dysbiosis contributes to neuropsychiatric outcomes through immune-mediated mechanisms [17]. Altered gut microbiome composition can impair gut barrier permeability, activate systemic inflammation and immune responses, and increase leakiness in the blood–brain barrier (BBB) leading to neuroinflammation. Data supporting the role of neuroinflammation in neuropsychiatric disorders including depression are extensive [30].

Given these findings and the identified knowledge gaps, we hypothesized that children with PANDAS harbor altered microbial communities across multiple body sites, including the nasal, oral and gut microbiomes, characterized by depletion of beneficial commensals and enrichment of pro-inflammatory pathobionts, and that these compositional shifts are accompanied by functional consequences including altered production of neuroactive metabolites, disrupted short-chain fatty acid and bile acid metabolism, and compromised gut barrier integrity, thereby contributing to immune dysregulation and neuropsychiatric symptoms via the gut–brain axis. To address these gaps, we performed an integrated multi-omics analysis of nasal, oral, and fecal samples from pediatric PANDAS cases and healthy controls, including discordant twin pairs, using 16S rRNA gene sequencing, shotgun metagenomics, and untargeted fecal metabolomics.

## 2. Materials and Methods

**Participants:** A total of 19 participants were classified into two groups: PANDAS (n = 10), and healthy controls (HC; n = 9), with the median age in the PANDAS group of 6.71 ± 3.9 years, and 11 (±4.6) years in healthy controls (Table 1). The PANDAS subjects were diagnosed at the University of Arizona Children’s Post-Infectious Autoimmune Encephalopathy (CPAE) clinic and met the diagnostic criteria for PANDAS, which include sudden onset or exacerbation of OCD and/or tic disorders following a streptococcal infection as well as associated secondary characteristics [6]. The control group had no history of neuropsychiatric or autoimmune disorders. All specimen collections were required to be scheduled at minimum 30 days after that last antibiotic dose.

**Sample collection:** Upon enrollment, each parent of a participant received a stool sample collection kit and instructions on how to collect samples and fill out the collection form. The kit included a prelabeled barcoded OMNIgene•GUT tube with a collection spatula (DNA Genotek, Stittsville, ON, Canada, cat. #: OMR-200), disposable collection tools [Commode Specimen Collection System for bulk stool sample collection (Fisher, Hampton, NH, USA, cat. # 02-544-208), powder-free nitrile gloves, and a visual step-by-step instruction sheet]. All samples were sent to the lab via FedEx or delivered to the clinic during a follow-up visit when nasal and oropharynx microbiome samples were also collected using OMNIgene•ORAL (DNA Genotek, cat. #: OMR-110). Samples were transported to the research lab and stored in −80 °C freezers until used.

**DNA extraction:** DNA was extracted with QIA PowerFecal pro DNA kit (QIAGEN, Germantown, MD, USA) according to the manufacturer’s instructions. Extraction blanks were included for contamination control during extraction, library preparation and sequencing.

**16S amplicon sequencing and data analysis:** To characterize potential differences in the nasal, oral, or gut microbiome between PANDAS and healthy control subjects, we performed amplicon sequencing. The V4 hypervariable region of the 16S rRNA gene was PCR-amplified using the 515-F and 806-R primer pair [31] with Illumina adapters and a unique 12 base pair (bp) barcode for each sample. PCR product was quantified with a Quant-iT PicoGreen dsDNA Assay Kit (Invitrogen, Carlsbad, CA, USA). DNA products were then pooled in equimolar concentration; products longer than 200 bp were selected using a QIAseq beads and sequenced on a 2 × 150 bp Illumina MiSeq platform (Illumina, San Diego, CA, USA) at the University of Arizona’s PANDA Core for Genomics and Microbiome Research. Demultiplexing was performed using idemp (https://github.com/yhwu/idemp, (accessed on 25 April 2025)). Raw paired-end 16S rRNA gene sequences were processed using the DADA2 pipeline [32] (version 1.26) in R (version 4.3). Reads were quality filtered (maxEE = 2, truncQ = 2), error corrected, and chimeras identified and removed, as implemented in the dada2 pipeline. The resulting amplicon sequence variants (ASVs) were assigned taxonomy using the SILVA reference database (version 138.1). All downstream analyses were performed in R using the phyloseq, vegan, microbiome, and DESeq2 packages. ASVs were filtered to retain those with a minimum detection threshold of 10 counts and present in at least 5% of samples. Alpha diversity was assessed using observed richness (number of ASVs) and Shannon diversity index calculated on raw ASV counts. Differences in alpha diversity were evaluated using Wilcoxon rank-sum tests. Beta diversity was assessed using Bray–Curtis dissimilarity on relative abundance-transformed data. Community composition differences between groups were visualized using Non-metric Multidimensional Scaling (NMDS). Statistical significance of community differences was evaluated using Permutational Multivariate Analysis of Variance (PERMANOVA [33]; adonis2 function, 999 permutations). Homogeneity of group dispersions was tested using the betadisper function to verify PERMANOVA assumptions. For differential ASV abundance testing, we used DESeq2 with the Wald test and local dispersion fitting, which is robust for small sample sizes. Size factors were estimated using the “poscounts” method to handle sparse microbiome count data. *p*-values were adjusted for multiple testing using the Benjamini–Hochberg false discovery rate (FDR) method. ASVs were considered statistically significant at FDR-adjusted *p* < 0.05 with |log_2_ fold change| > 1. All analyses were conducted in R (version 4.3) using the following packages: phyloseq (v1.42), vegan (v2.6), DESeq2 (v1.38), microbiome (v1.20), and ggplot2 (v3.4) for visualization.

### Subset Analysis: Twins Discordant for PANDAS

Three pairs of twins [one identical (female–female) and two fraternal (female–female, and female–male)] were analyzed separately. Upstream data pre-processing was conducted as described above. Alpha diversity (observed richness, Shannon) was compared between PANDAS and control twins using paired Wilcoxon signed-rank tests. Within-pair differences were calculated for each twin pair and tested against a null hypothesis of zero difference. Beta diversity was assessed using Bray–Curtis dissimilarity. Within-pair distances (between co-twins) were compared to between-pair distances (unrelated individuals) using Wilcoxon rank-sum tests to evaluate whether twins were more similar to each other than to unrelated individuals. Community composition differences were tested using PERMANOVA with permutations restricted within twin pairs (strata = twin_pair, 999 permutations). NMDS ordination plot includes lines connecting twin pairs to visualize within-pair relationships. For differential abundance testing, ASVs with zero counts were removed and a prevalence filter was applied to retain ASVs present in at least two samples. DESeq2 was used with a paired design formula (~twin_pair + diagnosis) to control for between-pair variation while testing the effect of diagnosis. The Wald test was applied with local dispersion fitting (fitType = “local”), which is robust for small sample sizes, and size factors were estimated using the “poscounts” method to handle sparse microbiome count data. *p*-values were adjusted using the Benjamini–Hochberg FDR method. ASVs were considered statistically significant at FDR-adjusted *p* < 0.05 with |log_2_ fold change| > 1.

**qPCR analysis of *Akkermansia muciniphila* abundance:** Relative abundance of *Akkermansia muciniphila* was analyzed by qPCR using primers targeting the 16S rRNA gene of *A. muciniphila* (forward primer 5′-CAGCACGTGAAGGTGGGGAC-3′ reverse primer 5′-CCTTGCGGTTGGCTTCAGAT-3′) [34] and normalized using pan-bacterial 16S primers (forward primer 5′-TCCTACGGGAGGCAGCAGT-3′, reverse primer 5′-GGACTACCAGGGATCTAATCCTGTT-3′) [35] (Sigma-Aldrich, St. Louis, MO, USA). Primers were used at a final concentration of 200 nM with SensiFAST™ SYBR^®^ No-ROX Kit (Meridian Bioscience, Swedesboro, NJ, USA) and Roche LightCycler96. The results were analyzed using the comparative Ct method as the means of relative quantification, normalized to pan-bacterial 16S as the housekeeping gene and relative to a calibrator (normalized Ct value obtained from healthy control subjects), and expressed as 2^−ΔΔCt^ (Applied Biosystems User Bulletin #2: Rev B “Relative Quantification of Gene Expression”). Mann–Whitney test was used for statistical comparison

**Shotgun metagenomic sequencing and data analysis:** A subset of samples (7 healthy controls and 6 PANDAS) was used for shotgun metagenomic sequencing and analysis. The metagenomic DNA libraries were constructed using genomic DNA isolated from specimens using Illumina DNA Prep kit according to the manufacturer protocol. The quality of all libraries was evaluated using an Agilent TapeStation 4150 with a DNA HS D1000 tapes. The shotgun sequencing was carried out on Illumina NextSeq550 paired-end 150 read length. Reads were first quality filtered, and adapter removed with trimmomatic v0.38 (LEADING:20 TRAILING:20 SLIDINGWINDOW:4:15 MINLEN:50) [36]. Host contaminants were removed by aligning sequences to the human reference genome *GRCh38* using bowtie2 [37].

Taxonomic profiling of shotgun metagenomic reads was performed using MetaPhlAn 4 (version 4.2.4) with the CHOCOPhlAn SGB database (mpa_vJan25_CHOCOPhlAnSGB_202503). Quality-filtered paired-end reads were processed with a minimum read length threshold of 70 bp. Individual sample profiles were merged to generate a unified relative abundance table. Species-level profiles were extracted by filtering taxonomic assignments containing species-level annotations while excluding strain-level assignments. Differential abundance analysis employed a three-tiered statistical approach to address the zero-inflated nature of microbiome data: (1) MaAsLin2 analysis—species present across samples were analyzed using the Microbiome Multivariable Associations with Linear Models package (MaAsLin2) in R. Data were log-transformed without additional normalization and linear models were fitted with diagnosis (PANDAS vs. control) as the fixed effect and control as the reference group. Significance was assessed at q < 0.25 (Benjamini–Hochberg FDR correction). (2) Presence/absence analysis: to identify species completely lost or gained in PANDAS patients, Fisher’s exact test was applied to 2 × 2 contingency tables comparing species presence/absence between groups. Species were classified as “Lost in PANDAS” (present in ≥1 control, absent in all PANDAS) or “Gained in PANDAS” (present in ≥1 PANDAS, absent in all controls). (3) Abundance analysis for shared species: for species detected in both groups, Wilcoxon rank-sum tests were performed to compare relative abundances. Log_2_ fold changes were calculated as log_2_ [(mean_PANDAS + 0.01)/(mean_Control + 0.01)] with a pseudocount to handle zeros. *p*-values were adjusted using the Benjamini–Hochberg method. All analyses were performed in R (version 4.5.2) using the following packages: MaAsLin2 for multivariable association testing, vegan for ecological statistics, and ggplot2, heatmap, and cowplot for visualization. Statistical significance was defined as *p* < 0.05 for individual tests, with FDR correction applied where appropriate.

*De novo* assembly was performed using megahit v1.2.9 [38]; contigs shorter than 500 bp were discharged. Prodigal v2.6.3 was used to predict protein-coding sequences [39]. Genes longer than 100 bp, from all samples were clustered at ≥95% identity and ≥90% overlap using MMseqs2 v13.45111 [40] resulting in a catalog containing 2,420,102 non-redundant genes. Paired-end reads were mapped onto the catalog with BWA v0.7.17-r1188 [41]. Functions were inferred by comparison to Kyoto Encyclopedia of Genes and Genomes (KEGG) database using Kofam scan v1.3.0 [42], resulting in 31.2% of genes with assigned to a KEGG Orthology (KO). Genetic and functional dissimilarities across samples were calculated with MaAsLin2 with standard settings. Bray–Curtis metrics on CSS-transformed tables. Omixer-RPM v1.0 [43] was used to group KEGGs into gut metabolic modules (GMM) and gut–brain modules (GBMs), which proposed functional pathways for the interaction of gene products, bacterial metabolism, and neural signaling in the gut environment. Binning was performed with the metaWRAP v1.3 pipeline [44]. First reads were assembled using megahit, then binning was performed, combining the results of three tools, MaxBin2, metaBAT2 and CONCOCT. Bins with completeness >70% and contamination <10% were used for analysis. The relative abundance of generated bins in each sample was calculated with coverM v0.6.1 (https://github.com/wwood/CoverM, (accessed on 25 April 2025)) [45]. GTDB-Tk v2.1.0 was used to assign taxonomy to each bin [46]. Within each bin, Prodigal v2.6, Kofam scan v1.3.0, and Omixer-RPM were used to infer KEGG modules and group them into GMMs and GBMs.

**Fecal untargeted metabolomics:** 17 fecal samples (9 from PANDAS subjects and 8 from healthy controls) were submitted for untargeted metabolomics analysis to Metabolon (Morrisville, NC, USA). Samples were prepared using the automated MicroLab STAR^®^ system from Hamilton Company (Reno, NV, USA). To remove protein, dissociate small molecules bound to protein or trapped in the precipitated protein matrix, and to recover chemically diverse metabolites, proteins were precipitated with methanol under vigorous shaking for 2 min (Glen Mills GenoGrinder 2000, Clefton, NJ, USA) followed by centrifugation. The resulting extract was divided into five fractions: two for analysis by two separate reverse phase (RP)/UPLC-MS/MS methods with positive ion mode electrospray ionization (ESI), one for analysis by RP/UPLC-MS/MS with negative ion mode ESI, one for analysis by HILIC/UPLC-MS/MS with negative ion mode ESI, and one sample was reserved for backup. Samples were placed briefly on a TurboVap^®^ (Zymark, Hopkinton, MA, USA) to remove the organic solvent. The sample extracts were stored overnight under nitrogen before preparation for analysis. A total of 991 named biochemicals were detected in fecal samples.

Initial data analysis included log transformation and imputation of missing values, if any, with the minimum observed value for each compound. Principal component analysis (PCA) and PERMANOVA were used to test for differences in metabolic profiles of controls and PANDAS fecal samples based on Euclidean distance. Differential abundance of metabolites was tested using Welch’s Two-Sample *t*-Test; *p*-values were corrected for multiple testing using the Benjamini–Hochberg false discovery rate (FDR) method. For presence/absence analysis, metabolites detected in at least one sample of one group but absent from all samples of the other group were identified, and Fisher’s exact test was used to test for differential detection between groups. Pathway-level enrichment was assessed by aggregating significant metabolites by sub-pathway annotation and formal pathway enrichment analysis was performed using Fisher’s exact test (one-sided) to identify metabolic pathways over-represented among differentially abundant metabolites. For each of the 114 sub-pathways, a 2 × 2 contingency table was constructed comparing the proportion of significant metabolites (Welch’s *t*-test *p* < 0.05) within the pathway versus all other pathways. Separate enrichment analyses were conducted for metabolites elevated and reduced in PANDAS. *p*-values were corrected for multiple testing using the Benjamini–Hochberg false discovery rate (FDR) method.

## 3. Results

### 3.1. 16S Amplicon Sequencing Reveal Altered Stool but Not Nasal or Oral Microbiome in PANDAS

**Figure 1:**
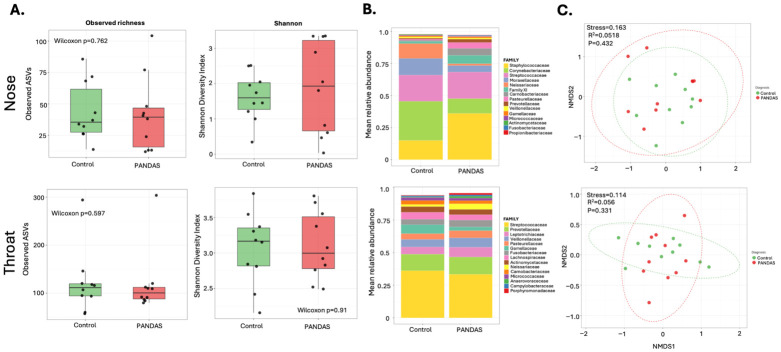
**Nasal and throat microbiome composition in PANDAS patients and healthy controls.** (**A**) Alpha diversity metrics for nasal and throat microbiome samples comparing PANDAS patients and controls. Observed richness (left) and Shannon diversity index (right) showed no significant differences between groups (Wilcoxon rank-sum test results shown in graph). Box plots display median, interquartile range, and individual data points. (**B**) Mean relative abundance of bacterial taxa at the family level in nasal and throat samples from controls and PANDAS patients. Stacked bar plots show community composition for each group. (**C**) Beta diversity analysis of nasal microbiome using Non-metric Multidimensional Scaling (NMDS) based on Bray–Curtis dissimilarity. No significant separation was observed between PANDAS and control groups (stress and PERMANOVA R^2^ and *p* values shown in graphs.

Nasal and throat swabs (n = 10 controls, n = 10 PANDAS), and stool samples (n = 9 controls, n = 10 PANDAS) were used for 16S amplicon sequencing. Differently sampled areas harbored clearly distinguished microbial communities (Supplemental Figure S1A–D). Analysis of nasal and throat samples revealed no statistical differences between healthy controls and PANDAS subjects in alpha diversity indices (richness or Shannon) or in beta diversity (Figure 1). Differential analysis of nasal microbial composition identified only three ASVs, all decreased in PANDAS; two of them belong to the *Corynebacterium* and *Streptococcus* genera (ASV2, P_adj_ = 7.74 × 10^−18^, ASV3 P_adj_ = 5.24 × 10^−21^) and one from the Neisseriaceae family (ASV31, P_adj_ = 8.89 × 10^−13^). Despite non-significant changes in diversity measures, the throat microbiome contained 11 differentially abundant ASVs, as shown in Supplemental Figure S1D.

**Figure 2:**
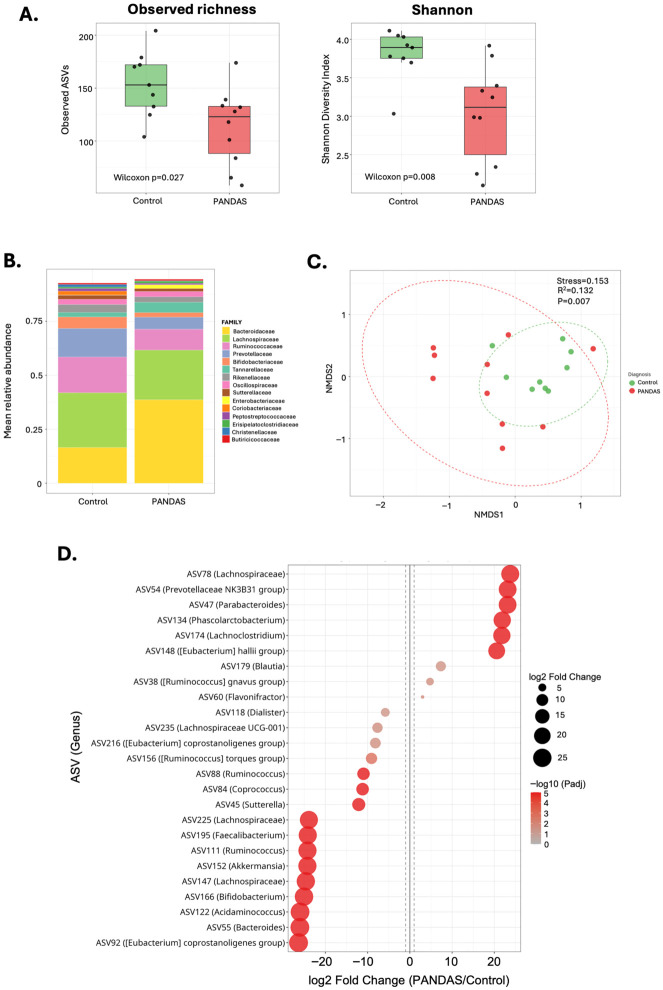
**Changes in alpha and beta diversity of the fecal microbiome dysbiosis in PANDAS patients compared to healthy controls.** (**A**) Alpha diversity measured by observed ASV richness and Shannon H’ index. PANDAS patients exhibited significantly reduced microbial richness compared to controls (Wilcoxon rank-sum test, *p* = 0.027 and *p* = 0.008, respectively). Box plots display median, interquartile range, and individual data points. (**B**) Mean relative abundance of bacterial taxa at the family level in fecal samples from controls and PANDAS subjects. (**C**) Beta diversity analysis using Non-metric Multidimensional Scaling (NMDS) based on Bray–Curtis dissimilarity. Significant separation was observed between PANDAS and control groups (PERMANOVA: R^2^ = 0.132, *p* = 0.007; stress = 0.153), indicating distinct community composition associated with PANDAS diagnosis. (**D**) Bubble plot depicting the top 25 differentially abundant ASVs between PANDAS subjects and controls. Bubble size and position indicate the magnitude and direction of log_2_ fold change, with taxa depleted in PANDAS shown on the left and taxa enriched in PANDAS shown on the right. One of the prominently reduced ASVs was ASV166, with 100% sequence homology with *Bifidobacterium bifidum* (log2FC = −25.12; P_adj_ = 1 × 10^−19^), a species with known probiotic effects capable of enhancing the epithelial barrier integrity [47]. ASV152 with 98.4% homology to *Akkermansia muciniphila* was also dramatically reduced (log2FC = −24.3; P_adj_ = 3.7 × 10^−16^). *A. muciniphila* is increasingly recognized for its influences host metabolism, strengthening of the gut barrier integrity, modulation of microbial composition and mucosal immune responses, in part due to production of short-chain fatty acids (SCFAs) [48]. Other notable SCFA producers decreased in PANDAS included ASV195 (99.2% identical with *Faecalibacterium butyricigenerans*; log2FC = −24.2, P_adj_ = 4.9 × 10^−15^), ASV84 (99.6% identical with *Coprococcus eutactus*; log2FC = −11.2, P_adj_ = 5.8 × 10^−7^), and two ASVs from the *Ruminococcus* genus, ASV88 and ASV111 (100% and 99.6% identity with *Ruminococcus callidus*, respectively). *Mediterraneibacter gnavus*, represented by ASV38 and known for its association with inflammatory bowel diseases (IBD), irritable bowel syndrome (IBS), Parkinson’s disease, neurocognitive disorders, and cognitive impairment, among others, was significantly increased (logFC = +4.8, P_adj_ = 0.022). Some of the ASVs that represent species with known beneficial effects on the host showed increased abundance, suggesting a compensatory response, e.g., ASV78 (100% identity with *Coprococcus phoceensis*), ASV47 (100% identity with *Parabacteroides distasonis*), or ASV60 (100% identity with *Flavonifractor plautii*).

However, stool samples from healthy controls and PANDAS subjects showed significantly lower richness (observed ASVs) and Shannon alpha diversity indices (Figure 2A), distinct compositional pattern at the family level (Figure 2B), and a significant difference in beta diversity as visualized in the NMDS plot (Figure 2C; PERMANOVA R^2^ = 0.132, *p* = 0.007). Differential abundance analysis of stool microbiota with strict criteria (P_adj_ < 0.05, log2 fold change >1) identified 26 ASVs (nine increased and 27 decreased in PANDAS), and 51 differentially abundant ASVs (12 increased and 39 decreased in PANDAS) with nominal criteria (raw *p* < 0.05) (Supplemental File S1). Figure 2D depicts the top 25 differentially abundant ASV in stool samples from PANDAS subjects.

### 3.2. Gut Microbiota in Twins Discordant for PANDAS

**Figure 3:**
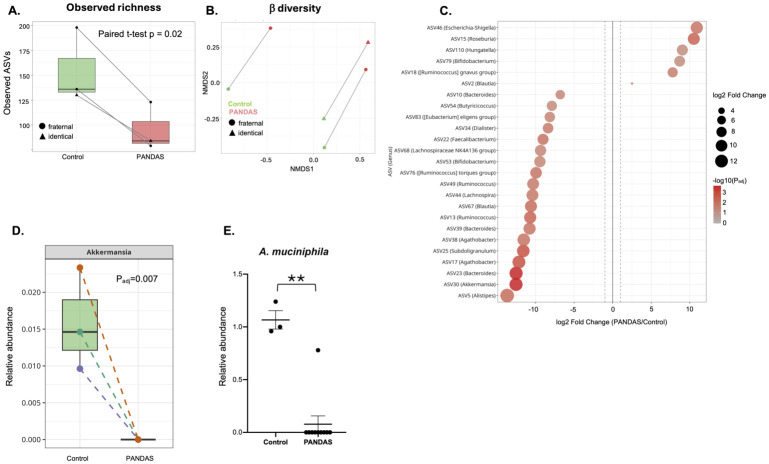
**Gut microbiome differences in twin pairs discordant for PANDAS.** (**A**) Alpha diversity comparison between PANDAS-affected twins and their healthy co-twins. (paired T test *p* = 0.02). (**B**) Beta diversity analysis showing NMDS ordination of PANDAS and healthy co-twin samples. (**C**) Top 25 differentially abundant ASVs between PANDAS-affected twins and healthy co-twins (P_adj_ < 0.05). Taxa depleted in PANDAS twins (left/blue) include *Akkermansia*, *Bacteroides*, *Faecalibacterium*, and *Agathobacter*. Taxa enriched in PANDAS twins (right/red) include *Escherichia-Shigella* and *[Ruminococcus] gnavus* group. (**D**) Change in the relative abundance of the ASV30 (Akkermansia) in each twin pair. (**E**) qPCR analysis of fecal samples from the PANDAS cohort using primers specific to *A. muciniphila* and normalized to 16S. ** *p* < 0.01.

Analysis of twins discordant for PANDAS controls for shared genetic background, early-life exposures, household environment, shared exposure to Group A Streptococcus (GAS), and dietary patterns, thereby isolating microbiome differences specifically attributable to the disease and its direct consequences (e.g., eating disorder) rather than other confounding host or environmental factors. Fecal microbiome was analyzed in a subset of the enrolled subjects, one pair of identical and two pairs of fraternal twins. As in previous analysis, we observed significant differences in richness (Figure 3A) and in beta diversity (Figure 3B). ASV-level analysis of differential abundance identified some of the same bacteria observed in all subjects although the ASV most significantly increased in PANDAS twins was ASV46 mapped to the *Escherichia-Shigella* genus containing several known pathogens and pathobionts, with 100% identity with *E. marmotae*, *E. fergusonii*, *E. coli* strain JCM 1649 serotype O1:K1:H7, *S. boydii*, *S. sonnei*, and *S. flexneri* (Figure 3C). ASV30, representing *A. muciniphila* was similarly dramatically decreased in abundance (log2FC = −13.24, P_adj_ = 0.007) (Figure 3D). This profound decline in relative abundance was conformed using real-time qPCR (Figure 3E). A total of 34 differentially abundant ASVs (10 increased and 24 decreased in PANDAS) with nominal criteria (raw *p* < 0.05) are listed in Supplemental File S2.

### 3.3. Shotgun Metagenomics

**Figure 4:**
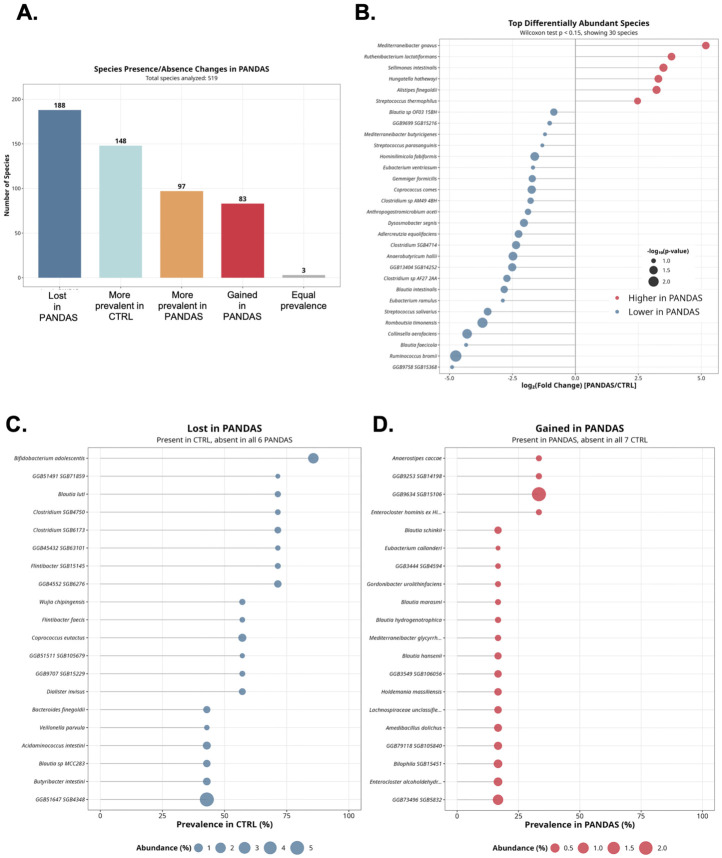
**Shotgun metagenomic profiling reveals widespread species loss in PANDAS gut microbiome.** (**A**) Summary of species-level differential abundance analysis. Species were categorized based on presence/absence patterns and prevalence differences between PANDAS patients and healthy controls lost in PANDAS (present in controls only), more prevalent in CTRL, equal prevalence, more prevalent in PANDAS, and gained in PANDAS (present in PANDAS only). The analysis reveals a marked asymmetry with substantially more species depleted than enriched in PANDAS. (**B**) Top differentially abundant species identified by MetaPhlAn 4 taxonomic profiling. Dot plot shows species with significant abundance differences between groups, with effect size indicating the magnitude and direction of change. (**C**) Species completely lost in PANDAS patients (detected in ≥1 control but absent in all PANDAS samples). Fisher’s exact test was used to assess the significance of presence/absence patterns. Notable losses include beneficial taxa associated with gut health and short-chain fatty acid production. (**D**) Species gained in PANDAS patients (detected in ≥1 PANDAS sample but absent in all controls). These taxa, though fewer in number than species lost, may contribute to the altered functional capacity of the PANDAS gut microbiome. All shown passed Fisher’s exact test at *p* < 0.05.

A total of 13 stool specimens from healthy controls (n = 7) and subjects with PANDAS (n = 6) were used for shotgun metagenomic analysis, with 815,3731 to 32,086,311 microbial reads per sample (Wilcoxon-test W = 15, *p* = 0.44, PANDAS vs. control). Alpha diversity indices (species richness and Shannon index) showed downward trends in PANDAS, albeit without reaching statistical significance (*p* = 0.054 and 0.13, respectively; Supplemental Figure S2A). However, beta diversity analysis showed clear sample separation based on diagnosis (*p* = 0.03, Supplemental Figure S2B). A total of 188 species detected in healthy controls were completely absent in PANDAS, while 83 species were uniquely present in PANDAS (Figure 4A). Figure 4B depicts top 30 differentially abundant species, and top 20 species lost or gained in PANDAS are shown in Figure 4C,D). The complete list and statistics of species-level analysis can be found in Supplemental File S3.

Shotgun metagenomic analysis revealed a pronounced asymmetry in the PANDAS-associated gut microbiome, with substantially more species lost or decreased than gained or increased (Figure 4A). Among the most notable losses, *Bifidobacterium adolescentis*, a species with well-documented immunomodulatory and barrier-protective properties [49], was detected in 6/7 controls but entirely absent from all PANDAS recipients (Fisher’s exact test *p* = 0.005). Multiple butyrate-producing *Firmicutes* were similarly depleted, including *Coprococcus eutactus*, *Coprococcus comes* (*p* < 0.05), *Anaerobutyricum hallii* (*p* < 0.05), and *Ruminococcus bromii* (1/6 vs. 7/7, *p* = 0.005), a keystone species in resistant starch degradation that cross-feeds other butyrate producers [50]. *Dysosmobacter segnis* (*p* < 0.05), *Adlercreutzia equolifaciens* (*p* < 0.05), and *Collinsella aerofaciens* (*p* < 0.05) were also significantly reduced. The depletion of these taxa, many of which contribute to short-chain fatty acid production, epithelial barrier maintenance, and immune homeostasis, is consistent with a loss of colonization resistance that may facilitate the expansion of the opportunistic pathogens and pathobionts described below.

**Figure 5:**
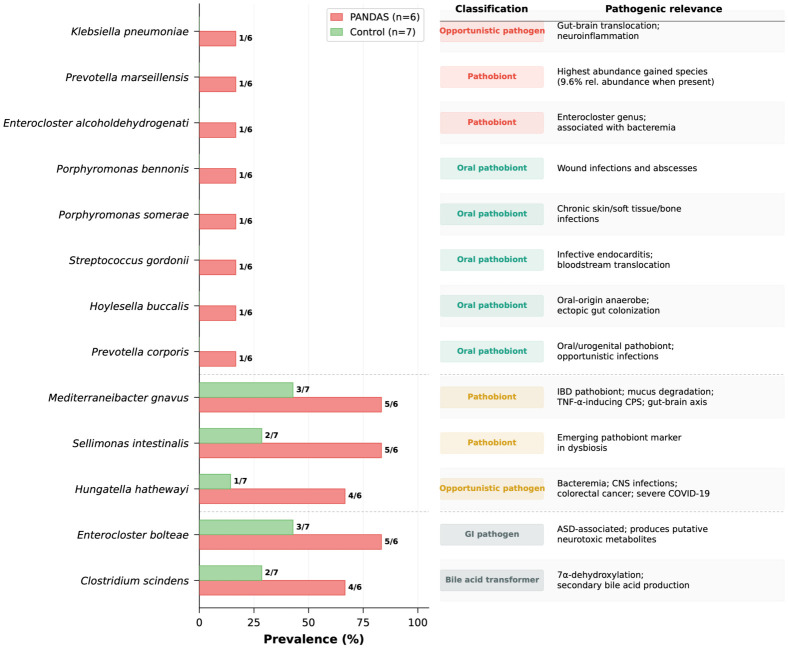
**Pathobionts and opportunistic pathogens enriched in the PANDAS gut microbiome**. Prevalence (% of subjects with detection) of species classified as recognized or potential pathobionts among those enriched in PANDAS-colonized mice (n = 6) compared to healthy controls (n = 7) by shotgun metagenomics. Species are grouped into three categories separated by dashed lines: gained in PANDAS (detected exclusively in PANDAS recipients and absent from all controls); increased (present in both groups but significantly more prevalent and abundant in PANDAS, Wilcoxon rank-sum test *p* < 0.05); and more prevalent (higher prevalence in PANDAS without reaching statistical significance). Numbers on bars indicate detection frequency (subjects detected/total). Classification badges indicate the pathogenic character of each species based on published literature.

To evaluate the pathogenic potential of species enriched in PANDAS, we examined the “Gained in PANDAS” and “More prevalent in PANDAS” species lists for recognized or potential opportunistic pathogens and pathobionts (Figure 5; Supplemental File S3). Among the 83 species detected exclusively in PANDAS recipients, several with documented pathogenic potential were identified. Most notably, *Klebsiella pneumoniae*, a well-established opportunistic pathogen recently shown to translocate from the gut to the brain and trigger neuroinflammation in mouse models [51], was detected in one PANDAS recipient (1/6) and absent from all controls. Additional species gained in PANDAS included *Porphyromonas bennonis*, *P. somerae*, *Streptococcus gordonii*, *Hoylesella buccalis*, and *Prevotella corporis*—taxa typically associated with the oral cavity or urogenital tract whose fecal detection suggests ectopic gut colonization via oral–gut translocation, consistent with disrupted barrier function. Among species present in both groups, three pathobionts showed significantly increased prevalence in PANDAS (Wilcoxon *p* < 0.05): *Mediterraneibacter gnavus* (formerly *Ruminococcus gnavus*; 5/6 vs. 3/7), a recognized IBD pathobiont that degrades intestinal mucus and produces an inflammatory capsular polysaccharide capable of inducing TNF-α from dendritic cells [52]; *Hungatella hathewayi* (4/6 vs. 1/7), an emerging opportunistic pathogen implicated in bacteremia, CNS infections, and colorectal cancer [53]; and *Sellimonas intestinalis* (5/6 vs. 2/7). *Enterocloster bolteae* (5/6 vs. 3/7), a gastrointestinal pathogen frequently enriched in children with autism spectrum disorder that produces putative neurotoxic metabolites [54], and *Clostridium scindens* (4/6 vs. 2/7), a bile acid 7α-dehydroxylating bacterium whose expansion is consistent with the elevated secondary bile acids observed in PANDAS fecal metabolomes, were also more prevalent in PANDAS recipients. Collectively, these findings indicate that the PANDAS-associated gut microbiome is not merely depleted of beneficial taxa but is actively enriched in opportunistic pathogens and pathobionts, including several with documented neuroinflammatory potential and others whose ectopic gut presence is indicative of compromised barrier integrity.

**Figure 6:**
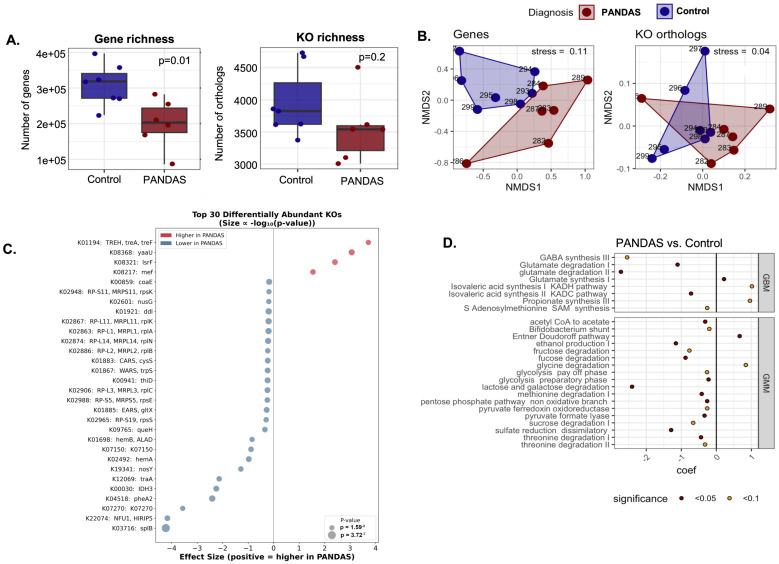
**Functional profiling of the gut microbiome by shotgun metagenomics reveals reduced metabolic capacity in PANDAS.** (**A**) Functional alpha diversity comparing PANDAS patients and healthy controls. Gene richness (left) and KEGG Ortholog (KO) richness (right) show reduced functional diversity in PANDAS gut microbiomes. (**B**) Beta diversity analysis using NMDS based on Bray–Curtis dissimilarity of gene profiles (left) and KO profiles (right). Ordination plots demonstrate separation between PANDAS and control groups, indicating distinct functional community composition. (**C**) Top 30 differentially abundant KEGG Orthologs between PANDAS patients and controls identified by MaAsLin2 analysis. The marked asymmetry shows predominantly depleted functions in PANDAS (141 KOs depleted vs. five enriched at *p* < 0.05 and |coefficient| > 1.0). (**D**) gut–brain modules (GBMs) and gut metabolic modules (GMMs) identified by Omixer-RPM analysis.

PANDAS cases exhibited a significantly lower number of non-redundant genes compared to controls (Welch’s *t*-test t (9.9) = −3.06, *p* = 0.01, Figure 6A). This reduction corresponded to a trend towards decreased KEGG Ortholog (KO) module potential in PANDAS subjects, though the difference was not significant (Welch’s *t*-test t (10.7) = −1.36, *p* = 0.2, Figure 6A). PERMANOVA analysis indicated a statistically significant difference in gene composition among microbial communities (R^2^ = 0.13, *p* = 0.03, Figure 6B), and marginal effect of their functional profiles (R^2^ = 0.17, *p* = 0.08, Figure 6B). Differential KO abundance analysis revealed a striking asymmetry: 141 KOs were significantly depleted in PANDAS compared to only five enriched (*p* < 0.05, |coefficient| > 1.0), suggesting widespread functional loss rather than gain of specific pathways (Figure 6C, complete list in Supplemental File S4). The results suggested (1) a gut–brain axis disruption through potential reduction in phenylalanine biosynthesis (pheA2), which cold limits the availability of aromatic amino acids that serve as precursors for dopamine and norepinephrine; (2) depletion of spore-forming bacteria (splB marker), which aligns with a decrease in butyrate-producing *Clostridia* (16S data); (3) altered bacterial communication through increased lsrF involved in AI−2 degradation and regulation of quorum sensing; (4) metabolic dysfunction, including TCA cycle impairment (IDH3), reduced expression of energy metabolism genes, and iron–sulfur cluster deficiency (NFU1) which could impact redox homeostasis; (5) activation of stress response suggested by increased trehalase (trehalose is a bacterial stress protectant and its elevated degradation may indicate chronic stress conditions in the gut environment). Modular analysis conducted with GBMs and GMMs revealed statistically significant differences between PANDAS subjects and controls (Figure 6D), including GBMs underrepresented in PANDAS that suggest reduced capacity for GABA synthesis and glutamate degradation (Supplemental File S5).

**Figure 7:**
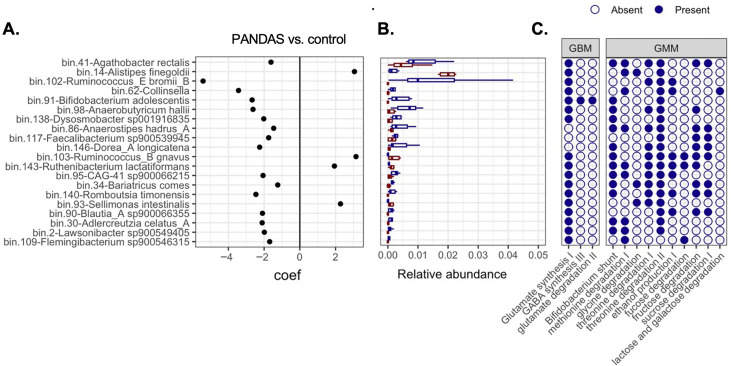
**Genome-level differences in fecal metagenomes**. (**A**) Metagenome-assembled genomes (MAGs) that are significantly different in abundance between PANDAS and control groups (*p* < 0.05). (**B**) The same expressed as change in relative abundance. (**C**) Contribution of differentially abundant MAGs to GBM and GMMs.

To assess genome-level shifts in microbial community structure, we performed contig binning to generate metagenome-assembled genomes (MAGs). A total of 158 MAGs were recovered, of which 55 met high standards criteria (Supplemental Figure S3A). These MAGs represented 148 species and 93 genera across nine major phyla (Supplemental Figure S3B), with sample-specific mapping rate ranging from 25% to 65%. Differential abundance analysis revealed four MAGs significantly enriched in PANDAS cases and 16 enriched in controls (Figure 7A,B, Supplemental File S6). Community composition and differential abundance patterns based on MAG relative abundances were consistent with those obtained using the database reference tool MetaPhlAn4. Functionally, the differentially abundant MAGs contributed to distinct GMBs and GMMs, with relevant KEGG orthologs localized within their genomes (Figure 7C).

### 3.4. Fecal Metabolomics

**Figure 8:**
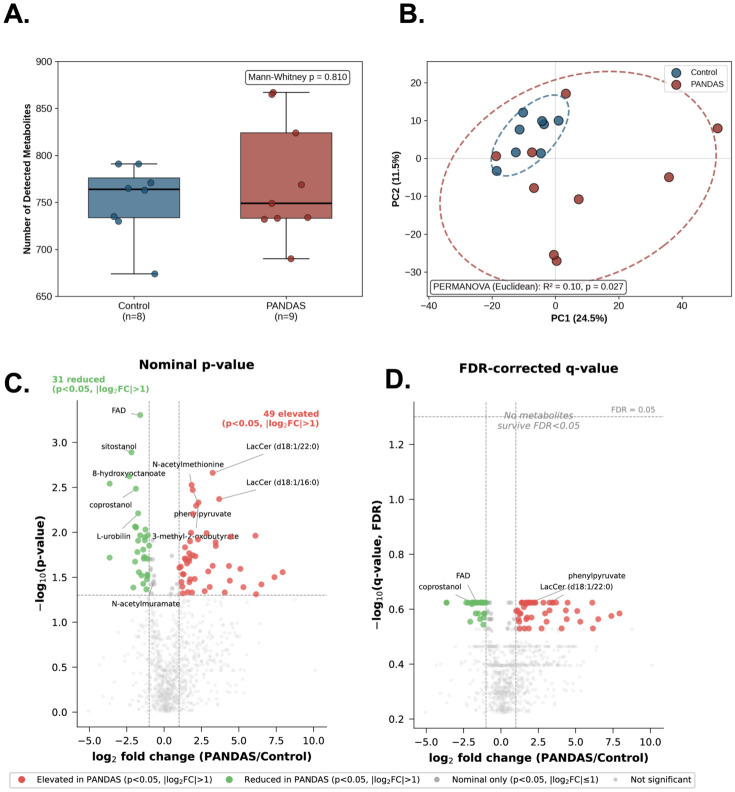
**Untargeted metabolomics reveals altered metabolite profiles in PANDAS subjects.** (**A**) Metabolic richness (number of detected metabolites) in control (n = 8) and PANDAS (n = 9) fecal samples. Boxes indicate interquartile range with median line; whiskers extend to 1.5× IQR. No significant difference was observed (Mann–Whitney U test, *p* = 0.81). (**B**) Principal component analysis (PCA) of fecal metabolite profiles. Each point represents one sample; ellipses indicate 95% confidence intervals. PERMANOVA analysis revealed significant separation between groups (R^2^ = 0.10, *p* = 0.025, Euclidean distance, 999 permutations). (**C**) Volcano plot displaying log_2_ fold change (PANDAS/control) against −log_10_ (nominal *p*-value) for 991 detected metabolites. Colored points indicate metabolites with nominally significant differential abundance (*p* < 0.05) and absolute log_2_ fold change >1: red = elevated in PANDAS (n = 49), green = reduced in PANDAS (n = 31). Dark gray points represent metabolites reaching nominal significance but with |log_2_FC| ≤ 1; light gray points are not significant. Dashed horizontal line indicates *p* = 0.05; dashed vertical lines indicate |log_2_FC| = 1. Selected metabolites are labeled, including FAD (the most statistically significant individual metabolite, *p* = 0.0005), bacterial biomass markers (coprostanol, L-urobilin, N-acetylmuramate), lactosylceramides (LacCer), and branched-chain amino acid intermediates. (**D**) The same data plotted against −log_10_ (FDR-corrected q-value, Benjamini–Hochberg method). No individual metabolites survived FDR correction at q < 0.05 (dashed horizontal line), consistent with the limited statistical power of the cohort (n = 9 PANDAS, n = 8 controls). Despite the lack of individual metabolite-level FDR significance, pathway-level enrichment analyses achieved FDR-corrected significance for monohydroxy fatty acids (P_adj = 0.00002), lactosylceramides (P_adj = 0.001), dipeptides (P_adj = 0.018), and leucine/isoleucine/valine metabolism (P_adj = 0.018) (Supplemental File S8).

Out of 991 detected metabolites, 63 were detected only in PANDAS subjects. Three of them passed Fisher’s exact test at p_nom_< 0.05, and 6 at p_nom_ < 0.1 (Supplemental Figure S4 and Supplemental File S7). They did not pass the FDR correction, which is likely a reflection of low sample size. Of the 63 PANDAS-exclusive metabolites, 47 (75%) were detected in two or more PANDAS subjects and 18 (29%) in three or more, indicating that the majority are not driven by individual outliers. The asymmetry itself (63 PANDAS-exclusive vs. one control-exclusive) is highly unlikely under a stochastic null model (binomial *p* = 3.5 × 10^−18^) and is partly attributable to medication and dietary supplement use in the clinical cohort (nine drug metabolites and 20 food/plant-derived compounds), with the remainder representing endogenous or microbially derived metabolites clustering in biologically coherent pathways (Supplemental File S7). Lactosylceramide LacCer(d18:1/20:0) (HMDB0011593) was detected in 6/7 PANDAS subjects, and none of the controls (p_nom_ = 0.009). Interestingly, it is one of the products of β-1,4-galactosyltransferase 6 (B4GALT6) and has been reported to contribute to the activation of the CNS innate immune responses and neurodegeneration in experimental autoimmune encephalitis (EAE) [55] and glial activation [56,57]. Of interest, 2/7 PANDAS subjects showed detectable traces of amoxicillin (p_nom_ = 0.47), which suggest that despite the defined enrolment criteria (at least 30 days after last antibiotic dose), this may to some extent represent a confounding factor. Differential abundance analysis was conducted with batch-normalized, imputed, and log2-transformed data. While metabolic richness (number of detected metabolites) did not differ between groups (*p* = 0.81; Figure 8A), principal component analysis demonstrated significant separation of PANDAS and control metabolomes (PERMANOVA: R^2^ = 0.10, *p* = 0.025; Figure 8B), indicating altered metabolic profiles despite comparable metabolite diversity.

**Figure 9:**
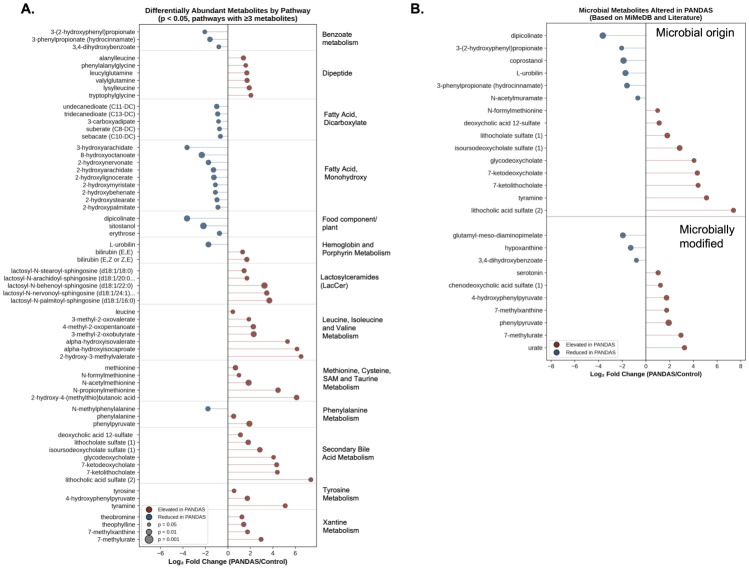
**Differentially abundant fecal metabolites and their classification.** (**A**) Dot plot of differentially abundant metabolites (*p* < 0.05) grouped by metabolic sub-pathway. Dot color indicates direction of change; dot size reflects statistical significance. Only pathways with ≥3 differentially abundant metabolites are shown. (**B**) Differentially abundant metabolites (*p* < 0.05) of microbial origin classified using MiMeDB and the literature. Dot color: direction of change (red = elevated, blue = reduced in PANDAS); dot size: statistical significance. Secondary bile acids (bacterial 7α-dehydroxylation products) were uniformly elevated, while bacterial biomass markers (coprostanol, L-urobilin, N-acetylmuramate, dipicolinate) were reduced, confirming altered microbial composition in PANDAS patients.

Differential abundance analysis identified 98 metabolites with nominally significant differences (*p* < 0.05), though none survived FDR correction (0.238 < q < 0.296), likely reflecting the modest sample size. Of these, 55 metabolites were elevated and 43 were reduced in PANDAS patients (Figure 8C; Supplemental File S7). These findings should be considered as hypothesis-generating and require validation in larger cohorts. Pathway-level aggregation of differentially abundant metabolites is depicted in Supplemental Figure S5. Figure 9A depicts differentially abundant metabolites in selected pathways (with ≥3 metabolites per pathway). Metabolites/pathways enriched in PANDAS included dipeptide, hemoglobin and porphyrin metabolism, lactosylceramides, leucine, isoleucine and valine metabolism, methionine, cysteine, SAM, and taurine metabolism, phenylalanine metabolism, secondary bile acid metabolism, tyrosine metabolism and xanthine metabolism. Among those reduced in PANDAS were metabolites associated with benzoate metabolism, fatty acids (dicarboxylate and monohydroxy), and food components/plants (Figure 9A, Supplemental File S7).

Of the 63 PANDAS-exclusive metabolites, the majority (47/63, 75%) were detected in two or more PANDAS subjects, and 18 (29%) in three or more (Supplemental File S9), indicating that the asymmetry is not driven by individual outliers. Functional categorization revealed that 29 of the 63 (46%) were exogenous in origin, comprising drug metabolites (n = 9; including sertraline, amoxicillin, ibuprofen, and cannabidiol) and food/plant-derived compounds (n = 20; predominantly piperine and isoflavone conjugates likely reflecting supplement use). The remaining 34 endogenous or microbially derived metabolites clustered into biologically coherent pathways, most notably bile acid metabolism (nine species, including taurine- and glycine-conjugated primary and secondary bile acids detected in 2–4/9 subjects), sulfur amino acid intermediates (DMTPA, 4-methylthio-2-oxobutanoate), and a lactosylceramide species (LacCer d18:1/20:0, 6/9 subjects). The pathway coherence of these exclusive metabolites and their concordance with independent microbiome findings argue against stochastic detection of low-abundance compounds.

Pathway-level analysis revealed consistent alterations across multiple metabolic categories, with notable enrichment of secondary bile acid metabolism (seven metabolites elevated), branched-chain amino acid metabolism (seven elevated), lactosylceramides (five elevated), and dipeptides (six elevated) in PANDAS. Conversely, fatty acid monohydroxy metabolites (nine reduced) and dicarboxylate fatty acids (five reduced) were depleted in PANDAS patients (Supplementary File S7). Statistical analysis of pathway enrichment agreed with these findings with reduced monohydroxy fatty acids (P_adj_ = 0.00002), and elevated lactosylceramides (P_adj_ = 0.00005), dipeptides (Padj = 0.018), and leucine, isoleucine, and valine metabolism (P_adj_ = 0.018) in PANDAS subjects. Among nominally significant (*p* < 0.05) but not FDR-corrected were secondary bile acid metabolism (increased in PANDAS; *p* = 0.002), methionine, cysteine, SAM, and taurine metabolism (increased in PANDAS; *p* = 0.007), and dicarboxylate fatty acids, (reduced in PANDAS, *p* = 0.004) (Supplementary File S8).

Given the observed microbiome compositional shifts, we investigated whether differentially abundant metabolites were of microbial origin by cross-referencing with the Human Microbial Metabolome Database (MiMeDB) and the published literature. Of the 98 differentially abundant metabolites, 25 (26%) were identified as having definitive microbial origin or undergoing significant microbial modification (Figure 9B). Strikingly, all seven differentially abundant secondary bile acids, products of bacterial 7α-dehydroxylation were elevated in PANDAS patients (Figure 9A,B). Secondary bile acids are exclusively produced by gut bacteria, particularly *Clostridium* species, from host-derived primary bile acids. This elevation suggests increased bacterial bile acid transformation activity. Conversely, several markers of beneficial bacterial activity were reduced in PANDAS patients, including coprostanol (bacterial cholesterol reduction), *L*-urobilin (bacterial bilirubin metabolism), *N*-acetylmuramate (bacterial peptidoglycan component), and dipicolinate (bacterial spore marker). The reduction in these microbially derived metabolites, combined with the species-level losses observed in metagenomic analysis, suggests a functional depletion of beneficial gut bacteria in PANDAS.

## 4. Discussion

Oral and upper respiratory tract microbiome alterations have been reported in disorders such as autism spectrum disorder and schizophrenia, and the results are heterogenous and often lack consistent taxonomic signatures [58]. In contrast, more reproducible microbiome shifts have been observed in the gut across multiple neuropsychiatric and neuroinflammatory conditions, including depression. Although the oral microbiome has been hypothesized to contribute to the pathogenesis of PANS based on evidence from other disorders [59], direct characterization has not previously been reported. Ours is the first report that includes evaluation of oral and nasal microbiota. Importantly, no bacterial taxa previously demonstrated to modulate immune responses in other settings were identified among the differentially abundant amplicon sequence variants (ASVs) in PANDAS subjects at these sites [60]. *Fusobacterium nucleatum*, a species known for its involvement in gingivitis, systemic inflammatory responses, and associations with Alzheimer’s disease [60], was not directly identified as a differentially abundant taxon in our cohort. However, a single *Fusobacterium* ASV134 detected in the throat microbiota was decreased in PANDAS. This ASV shared 99.6% identity with *F. periodonticum* and *F. pseudoperiodonticum*, suggesting close relatedness within the genus but not allowing definitive assignment to *F. nucleatum.* This distinction is important because it limits species-level interpretation of the finding and avoids overattributing known *F. nucleatum*-associated effects.

Since our integrated analysis using 16S rRNA amplicon sequencing, shotgun metagenomics, and untargeted metabolomics of fecal samples provided compelling evidence for distinct gut microbiome and metabolomic signatures in pediatric PANDAS patients, our focus was placed on the gut microbiota. Our findings were further supported by analyzing discordant twins, which controlled for shared genetics and early-life environment. This analysis provided strong evidence that the differences in the gut microbiome are associated with disease. 16S sequencing revealed significantly reduced gut microbial alpha-diversity (Richness and Shannon H’) and different community composition (beta-diversity) in PANDAS subjects compared to healthy controls, whereas nasal and throat microbial communities remained relatively unchanged. Reduced gut microbial diversity has been widely associated with impaired community resilience, disrupted microbial functions and a range of chronic conditions including acute diarrhea, inflammatory bowel disease (IBD), cancer, metabolic and neurodevelopmental disorders [61,62,63]. Key convergent findings included depletion of beneficial commensals (*Bifidobacterium*, *Akkermansia*, *Bacteroides*, *Ruminococcus*, *Faecalibacterium*) confirmed across 16S, shotgun, and MAG analyses. Examples of such concordance are provided by *Bifidobacterium* [reduced in 16S p_adj_ = 2.14 × 10^−19^), species-level analysis (*B. adolescentis* lost, *p* = 0.005), and MAGs (*p* = 0.011)], *Ruminococcus bromii* [reduced in 16S, shotgun (*p* = 0.004), and MAGs (*p* = 0.001)] or *Akkermansia* (reduced in both full cohort and discordant twins). Many of the affected taxa are major SCFA producers (butyrate, acetate), which are associated with gut barrier integrity and general immune homeostasis, and their depletion had been reported in other neuroinflammatory and autoimmune conditions [64]. Our findings thus indicate a site-specific pattern in PANDAS, with minimal detectable differences in oral and nasal microbiota but with significantly more pronounced alterations in the gut.

Both 16S sequencing and metagenomic analyses consistently demonstrated the enrichment of *Ruminococcus gnavus* in fecal specimens of PANDAS subjects. This species is frequently linked to pro-inflammatory states and implicated in various conditions including IBD, obesity, type II diabetes mellitus, and non-alcoholic fatty liver disease. Although its precise role in host pathology remains unclear, *R. gnavus* has been suggested to influence gut–brain axis signaling, potentially exacerbating neuroimmune interactions [65]. Beyond *R. gnavus*, systematic examination of species enriched in PANDAS revealed a broader pattern of pathobiont and opportunistic pathogen accumulation. In the Results section, we highlight *Klebsiella pneumoniae*, *Hungatella hathewayi*, *Enterocloster bolteae*, and *Clostridium scindens* as potential pathobionts. The enrichment of *E. bolteae* in PANDAS is particularly noteworthy given the clinical and immunological overlap between PANDAS and ASD and suggests potential shared microbiome-mediated pathogenic mechanisms across pediatric neuropsychiatric conditions.

We also observed an increased abundance of *Alistipes finegoldii*, a species whose genus has shown both protective effects (in colitis and ASD) and pathogenic associations (with anxiety, MDD, myalgic encephalomyelitis and chronic fatigue syndrome) [66]. An additional example of a species elevated in PANDAS is *Ruthenibacterium lactatiformans*, previously related to MDD [67,68]. Conversely, several beneficial commensals were depleted in PANDAS subjects, notably, *Ruminococcus bromii*, a keystone primary starch degrader [69], associated with production of SCFAs [70], and *Bifidobacterium adolescentis*, a known GABA producer [71], were significantly reduced. These results align with our metagenomic evidence for decreased abundance of genes involved in carbohydrate degradation and GABA biosynthesis pathways. Other depleted taxa included *Romboutsia timonensis* [72], the depletion of which has been previously linked to ASD, and *Collinsella*, associated with low-fiber diets [73] when depleted. Our findings underscore the role of altered microbial functions and immune signaling in PANDAS pathophysiology, supporting the concept that dysregulated host–microbiome interactions may drive disease progression.

While the dramatic depletion of *Bifidobacterium* exemplified by the loss of ASV166 (*B. bifidum*; log_2_FC = −25.12, P_adj_ = 1 × 10^−19^) in 16S data and the absence of *B. adolescentis* from all PANDAS subjects in metagenomic profiling might be expected to produce detectable losses in associated KEGG Orthologs, particularly the fructose-6-phosphate phosphoketolase (F6PPK; EC 4.1.2.22) that defines the bifid shunt, the characteristic carbohydrate fermentation pathway of the genus [74], community-level KO profiling has limited resolution for detecting genus-specific functional losses because multiple taxa contribute to individual orthologs. Metabolomic data, however, provided complementary evidence consistent with reduced *Bifidobacterium* activity: in addition to the reduced *N*-acetylmuramate, flavin adenine dinucleotide (FAD) was the most significantly reduced metabolite in the entire dataset (FC = 0.33, *p* = 0.0005), with flavin mononucleotide (FMN) also trending lower (FC = 0.41, *p* = 0.18), consistent with the loss of *Bifidobacterium* species that are recognized intestinal producers of riboflavin and its cofactors [75].

Reduced bacterial biomass markers (coprostanol, L-urobilin, N-acetylmuramate) and increased abundance of LsrF, a coenzyme A (CoA)-dependent thiolase that catalyzes the final step in the processing and degradation of the Autoinducer-2 (AI-2), a critical quorum sensing signal in bacteria such as *E. coli* and *S. enterica* [76], provide further evidence of systemic disruption of the gut microbial community structure PANDAS. Reduction in coprostanol (*p* = 0.0033), a metabolite exclusively produced by the *[Eubacterium] coprostanoligenes group* from cholesterol, was consistent with reduction in this taxon in 16S analysis (p_adj_ = 6.61 × 10^−17^). The reduction in gut microbial diversity was paralleled by a reduction in gene and functional (KO) richness in gut metagenomes of PANDAS subjects. KO findings that support reduced bacterial biomass and activity include reduced markers of protein synthesis (reduced rplK, rplA, rplN, rplC), decreased translational capacity (reduced tRNA synthetases), decreased peptidoglycan synthesis (reduced D-alanine-D-alanine ligase ddl), and decreased sporulation capacity (reduced spore photoproduct lyase splB). Despite the data suggesting decreased biomass, all seven differentially abundant secondary bile acids were elevated in PANDAS patients. Secondary bile acids are exclusively produced by bacterial 7α-dehydroxylation, primarily by *Clostridium* species in cluster XVIa. This may be attributable to an expansion of bile acid-transforming bacteria (*C. scindens*, *C. bolteae*) within a dysbiotic community, or potentially through altered enterohepatic circulation increasing substrate availability or via reduced bile acid reabsorption leading to increased colonic transformation. Although hypothetical at this point, increased production of secondary bile acids, through FXR- and TGR5-dependent signaling, may modulate systemic inflammation and immune responses, and can cross as well as affect the blood–brain barrier and interfere with neurotransmitter transporters (like dopamine, serotonin transporters), affecting mood and cognitive processes [77].

Our data provide potentially important insights into the altered gut–brain axis, especially via altered production of neuroactive metabolites. Fecal samples from PANDAS subjects showed reduced capacity for glutamate degradation and a concomitant increase in glutamate biosynthesis modules. While glutamate itself was not significantly altered in fecal metabolomics, these functional shifts may have downstream effects on neurotransmitter balance and excitatory signaling [78]. Similar disruptions in glutamate metabolism have been reported in ASD and psychiatric disorders [79,80]. Additionally, genes associated with gamma-aminobutyric acid (GABA) production were also diminished, a finding consistent with reduced inhibitory neurotransmitter status associated with pain modulation, and neurodevelopmental and behavioral regulation [80,81,82]. Elevated levels of neuromodulatory tryptamine (7.93-fold) and serotonin (2.03-fold; 5-hydroxytryptamine; 5-HT) are particularly relevant, given their known roles in gut motility, inflammation, and modulation of central neurotransmission. Tyramine, a bacterial metabolite of tyrosine, a trace monoamine with sympathomimetic properties (acts as a catecholamine-releasing agent) and TAAR1 receptor agonist, was increased 34-fold in PANDAS. Interestingly, we observed elevated fecal tryptamine and tyramine levels in GF mice colonized with *R. gnavus* [83], species significantly more abundant in our PANDAS cohort. Elevated tyramine has been associated with migraines, autonomic dysfunction, and altered mood symptoms, which are frequently reported in PANDAS. The finding of elevated tyramine alongside altered gut microbiome composition suggests a potential mechanism by which altered gut microbiome composition could contribute to neuropsychiatric symptoms through trace amine signaling.

Another interesting finding is related to increased fecal levels of lactosylceramides with d18:1 sphingoid base component. These compounds are likely of host origin and may indicate inflammatory stress within the gut epithelium. LacCer (d18:1/16:0) was suggested to be a reliable biomarker of intestinal inflammation in pediatric IBD [84] and of CNS inflammation in multiple sclerosis [85]. Lactosylceramide, synthesized by β-1,4-galactosyltransferase 6 (B4GALT6), has been implicated in neuroinflammation through activation of astrocytes via NF-κB and IRF-1 pathways, leading to production of pro-inflammatory chemokines including CCL2 and GM-CSF [55].

In the context of our findings, the gut–brain axis provides a compelling mechanistic framework linking microbiome alterations to neuroimmune dysfunction in PANDAS. Increasing evidence suggests that intestinal dysbiosis and altered microbial metabolite profiles can modulate systemic immune responses, promote intestinal permeability, and facilitate the generation of autoreactive antibodies. In PANDAS, where anti-neuronal antibodies are thought to play a central pathogenic role, such gut-derived immune activation may contribute to BBB disruption and subsequent neuroinflammation. Notably, analogous mechanisms have been well described in celiac disease, in which intestinal barrier dysfunction is associated with the development of anti-neuronal and anti-ganglioside antibodies and a spectrum of neurological manifestations [86,87]. These neurological gluten-related disorders represent a well-established model of immune-mediated crosstalk between the gut and the central nervous system. In line with this paradigm, our observation of altered gut microbiome composition and metabolite profiles in PANDAS patients supports the hypothesis that gut-derived immune and metabolic signals may contribute to disease pathophysiology. Future studies integrating microbiome, metabolomics, and immune profiling will be essential to further delineate causal pathways and identify potential therapeutic targets within the gut–brain axis.

Our study is not without limitations. PANDAS remains classified as a rare disease according to the Genetic and Rare Diseases Information Center (GARD:7312) and Orphanet (ORPHA:66624). Low prevalence limits the availability of suitable well-characterized subjects for research, resulting in our small sample size. Also, the absence of a specific ICD-10 code for PANDAS, often relying on proxies like D89.89 (other specified immune disorders), complicates epidemiological tracking and case identification in clinical databases, exacerbating challenges in assembling larger cohorts. It is plausible that the modest cohort size may have affected our ability to detect more subtle differences in the composition and metabolic activity of gut microbiota. Untargeted metabolomics results should be interpreted as hypothesis-generating, since none of the metabolites which reached nominal significance (*p* < 0.05) passed the FDR correction. Similarly, individual PANDAS-exclusive metabolites detected at low prevalence (1–2 subjects) should be interpreted with caution; however, the strong pathway-level coherence of these metabolites (e.g., nine bile acid species, all exclusive to PANDAS) and their convergence with independent microbiome findings support biological rather than stochastic origins. Detection of amoxicillin in 2/7 PANDAS subjects, suggests that antibiotic history was not always adequately reported by parents and may to a small extent represent a confounding factor. Cross-sectional design limits causal inference and future work should focus on longitudinal studies with larger cohorts tracking the microbiome during flares vs. remission. Some features of the PANDAS-associated microbiome particularly reduced alpha diversity and depletion of *Bifidobacterium* and butyrate-producing *Firmicutes* overlap with signatures reported in other pediatric immune-mediated conditions including IBD [88], juvenile idiopathic arthritis [89], or type 1 diabetes [90], and may therefore reflect a shared consequence of immune activation rather than PANDAS-specific pathology. Future studies incorporating autoimmune disease control groups matched for age and immunological burden will be important for delineating PANDAS-specific microbiome features from those common to pediatric autoimmunity more broadly. Finally, it is important to acknowledge that the reported changes in the gut microbiota likely represent the combined effect of the initial infection, extensive and varying treatment including antibiotics, as well as changed in dietary habits. Indeed, parents of all PANDAS patients in our cohort reported varying degrees of restricted and/or selective eating. Our approach was not to treat these factors as confounders, but rather to consider them as components of the overall clinical presentation, under the assumption that they converge to contribute to chronic modulation of brain function.

With these limitations in mind, our work nevertheless demonstrates that children with PANDAS exhibit distinct microbial and metabolic differences relative to healthy controls, potentially contributing to altered gut–brain axis signaling, consistent with reports that linked changes in gut microbiome and dysregulated microglial activity with various neurodevelopmental, neurobehavioral, and neurodegenerative disorders [16,91,92,93]. Given the critical role of the microbiome in host immunity, neurodevelopment, and metabolism, understanding the nature and functional consequences of altered gut microbiota in pediatric neuropsychiatric disorders remains an important research priority. While microbiome-targeted interventions have shown promise in other conditions associated with gut dysbiosis, their potential applicability to PANDAS requires validation through mechanistic studies such as fecal microbiome transplant into germ-free mice to address causality followed by appropriately designed clinical trials.

## Figures and Tables

**Table 1:** **Basic demographic information about the study participants**. Sex: M—male, F—female; Ethnicity: NHW—Non-Hispanic White, NHW/BAA—Non-Hispanic White/Black or African American, HW—Hispanic White, NHW/AI/AN—Non-Hispanic White/American Indian/Alaskan Native.

Diagnosis	Total Enrolled	Sex		Ethnicity				Age of Onset (Median ± SD)	Age at Consent (Median + SD)	Dietary Habits
		M	F	NHW	NHW/ BAA	HW	NHW/AI/ AN			
Control	9	3	6	3	1	4	1	N/A	11 (4.6)	Not recorded
PANDAS	10	2	8	8	1	0	1	6 (3.9)	6.71 (3.9)	Varying degree of restrictive/ selective eating

## Data Availability

Sequences are available in the NIH/NCBI data repository with BioProject ID PRJNA1405777.

## Supplementary Materials

The following supporting information can be downloaded at: https://www.mdpi.com/article/10.3390/microorganisms14051036/s1, Supplemental Figure S1. Body site-specific microbiome composition and oral differential abundance. (A,B) Alpha diversity (observed richness and Shannon index) differs significantly across fecal, nasal, and throat sampling sites (Kruskal–Wallis, *p* < 10^−5^). (C) NMDS ordination confirms distinct community composition by body site (PERMANOVA: R^2^ = 0.109, *p* = 0.001). (D) Mean relative abundance of top 15 bacterial families by site. (E) Differentially abundant ASVs in oral samples between PANDAS and controls, showing enrichment of *Leptotrichia* and depletion of commensal taxa in PANDAS patients. Supplemental Figure S2. Shotgun metagenomic diversity analysis of fecal samples from PANDAS patients and healthy controls. Thirteen fecal samples from healthy controls (n = 7) and PANDAS patients (n = 6) were analyzed by shotgun metagenomic sequencing, yielding 8.15 × 10^6^ to 3.21 × 10^7^ microbial reads per sample with no significant difference in sequencing depth between groups (Wilcoxon test, W = 15, *p* = 0.44). (A) Alpha diversity at the species-level. Species richness (left) and Shannon diversity index (right) showed downward trends in PANDAS patients compared to controls, approaching but not reaching statistical significance (*p* = 0.054 and *p* = 0.13, respectively; Wilcoxon rank-sum test). (B) Beta diversity analysis using Non-metric Multidimensional Scaling (NMDS) based on species-level Bray–Curtis dissimilarity. Significant separation was observed between PANDAS and control samples (PERMANOVA: R^2^ = 0.15, *p* = 0.03; stress = 0.11), indicating distinct metagenomic community composition associated with PANDAS diagnosis. Supplemental Figure S3: Contig binning to generate metagenome-assembled genomes (MAGs). (A) Completeness and contamination (both as percent) plot of extracted MAGs. (B) Phylogenomic tree of extracted MAGs. Three was calculated using phylophlan3 with standard settings. Supplemental Figure S4: Presence/absence analysis of fecal metabolites. (A) Distribution of metabolites detected exclusively in PANDAS or control samples, categorized by metabolic pathway. (B) Metabolites uniquely detected in PANDAS patients (Fisher’s exact test), including methionine pathway intermediates (4-methylthio-2-oxobutanoate, DMTPA), bile acid conjugates (glycochenodeoxycholate 3-sulfate), and lactosylceramides, supporting altered sulfur amino acid and sphingolipid metabolism in PANDAS. Of the 63 PANDAS-exclusive metabolites, 75% were detected in ≥2 subjects. Supplemental Figure S5: Pathway enrichment analysis of differentially abundant metabolites. Metabolic pathways significantly enriched among altered metabolites in PANDAS patients compared to controls. Key enriched pathways include branched-chain amino acid metabolism, methionine/sulfur amino acid metabolism, sphingolipid metabolism, and bile acid pathways, reflecting the functional consequences of altered gut microbiome composition on host metabolism. Supplemental File S1. 16S: Differential abundance analysis of stool microbiota. Supplemental File S2. 16S: Differential abundance analysis of the gut microbiota in twins discordant for PANDAS. Supplemental File S3. Shotgun metagenomics: Combined results from presence/absence and abundance analyses comparing gut microbiome species between PANDAS patients and healthy controls. Supplemental File S4. Shotgun metagenomics: Differential KEGG Ortholog (KO) abundance analysis in PANDAS patients and healthy controls. Supplemental File S5. Shotgun metagenomics: Functional pathway analysis of gut-axis using gut metabolic modules (GMM) and gut–brain modules (GBM) profiling. Supplemental File S6. Shotgun metagenomics: analysis of metagenome-assembled genomes (MAGs). Supplemental File S7. Fecal untargeted metabolomics data. Supplemental File S8. Fecal untargeted metabolomics: pathway enrichment.
