# Supplementary material for: Functional Shifts in Gut Microbiota and Associated Metabolites Suggest Gut–Brain Axis Dysregulation in Pediatric Autoimmune Neuropsychiatric Disorders Associated with Streptococcal Infections (PANDAS)
Source: Microorganisms. 2026 May 2;14(5):1036. doi: 10.3390/microorganisms14051036 (PMC13209760; doi:10.3390/microorganisms14051036)
Supplement: Supplementary file 1 [file microorganisms-14-01036-s001.zip › Supplemental Figures.pdf]

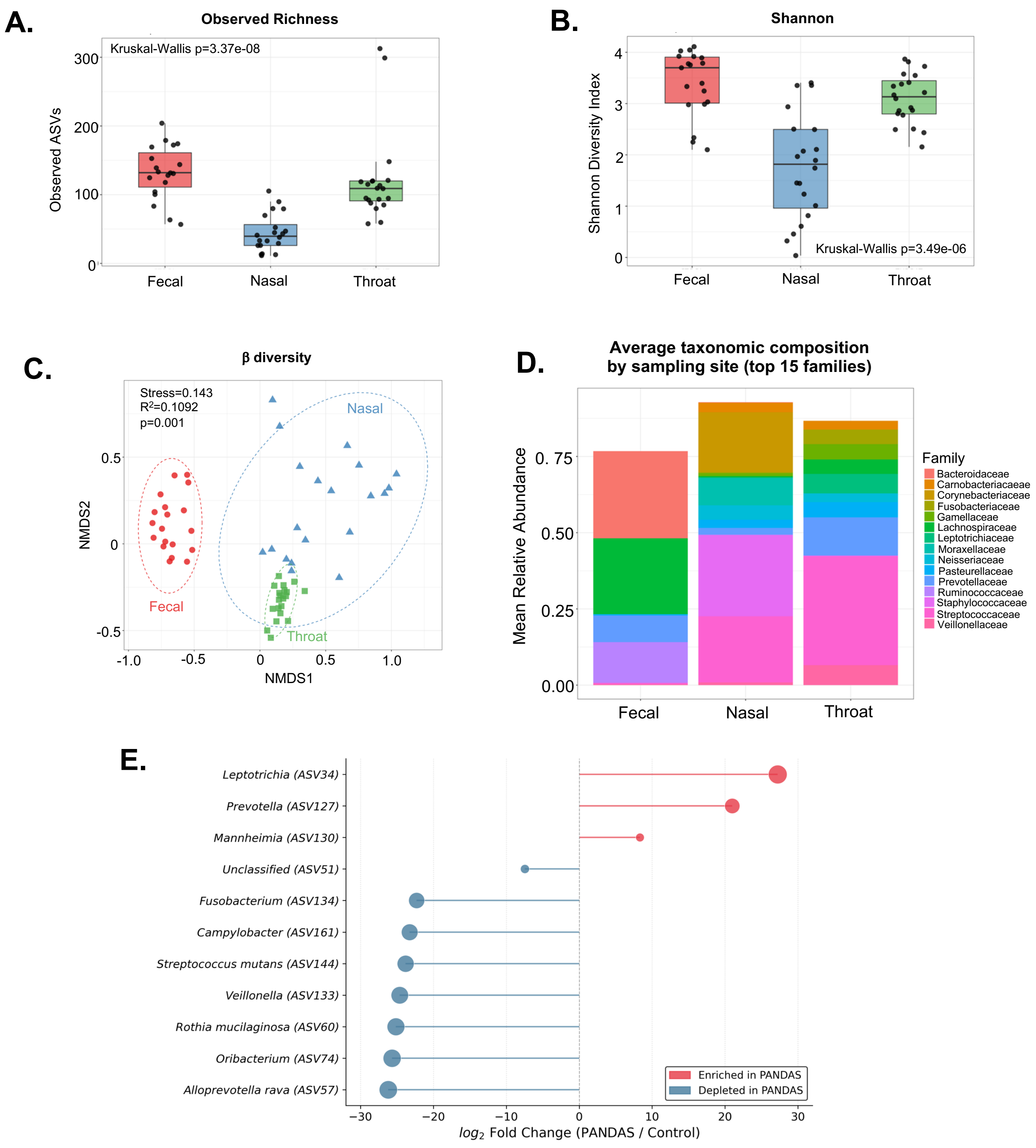

Supplemental Fig. S1: Body site-specific microbiome composition and oral differential abundance. (A–B) Alpha diversity (observed richness and Shannon index) differs significantly across fecal, nasal, and throat sampling sites (Kruskal-Wallis, $p < 10^{-5}$). **(C)** NMDS ordination confirms distinct community composition by body site (PERMANOVA: $R^2 = 0.109$, $p = 0.001$). **(D)** Mean relative abundance of top 15 bacterial families by site. **(E)** Differentially abundant ASVs in oral samples between PANDAS and controls, showing enrichment of *Leptotrichia* and depletion of commensal taxa in PANDAS patients.

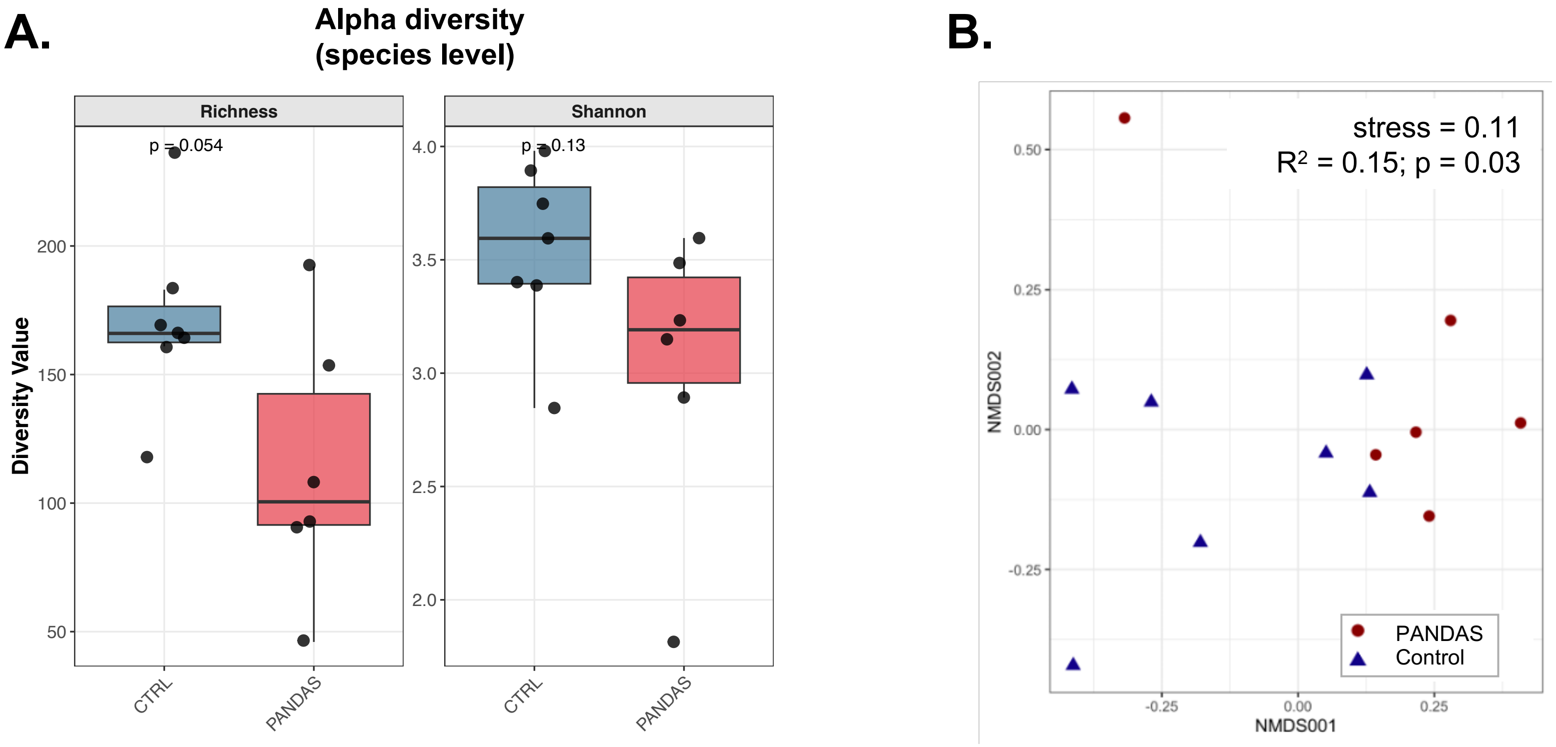

Supplemental Fig. S2: Shotgun metagenomic diversity analysis of fecal samples from PANDAS patients and healthy controls. Thirteen fecal samples from healthy controls (n = 7) and PANDAS patients (n = 6) were analyzed by shotgun metagenomic sequencing, yielding 8.15×10^6 to 3.21×10^7 microbial reads per sample with no significant difference in sequencing depth between groups (Wilcoxon test, W = 15, p = 0.44). **(A)** Alpha diversity at the species level. Species richness (left) and Shannon diversity index (right) showed downward trends in PANDAS patients compared to controls, approaching but not reaching statistical significance (p = 0.054 and p = 0.13, respectively; Wilcoxon rank-sum test). **(B)** Beta diversity analysis using Non-metric Multidimensional Scaling (NMDS) based on species-level Bray-Curtis dissimilarity. Significant separation was observed between PANDAS and control samples (PERMANOVA: R^2 = 0.15, p = 0.03; stress = 0.11), indicating distinct metagenomic community composition associated with PANDAS diagnosis.

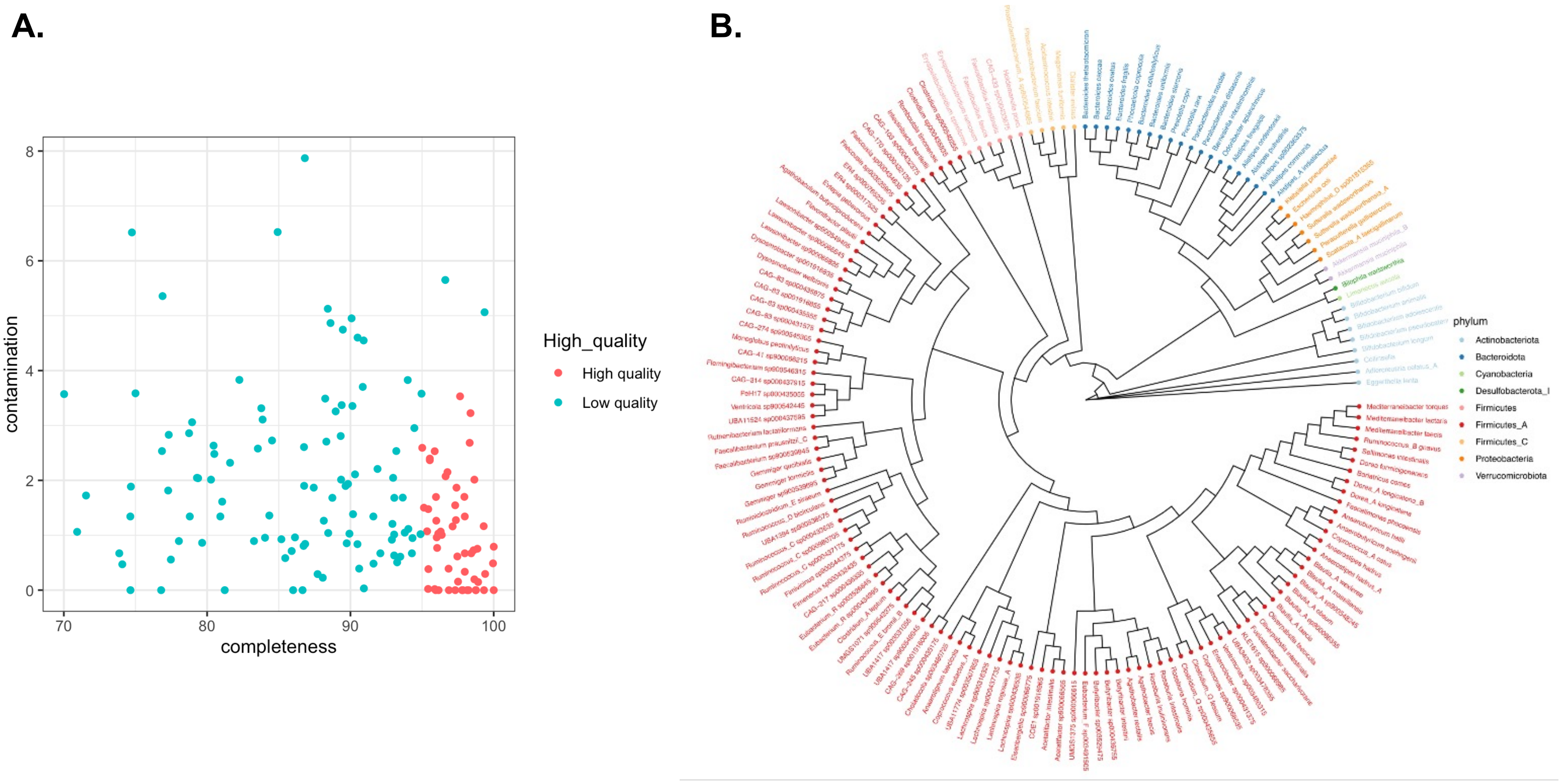

Supplemental Fig. S3: Contig binning to generate metagenome assembled genomes (MAGs). **(A)** Completeness and contamination (both as percent) plot of extracted MAGs. **(B)** Phylogenomic tree of extracted MAGs. Three was calculated using phylophlan3 with standard settings.

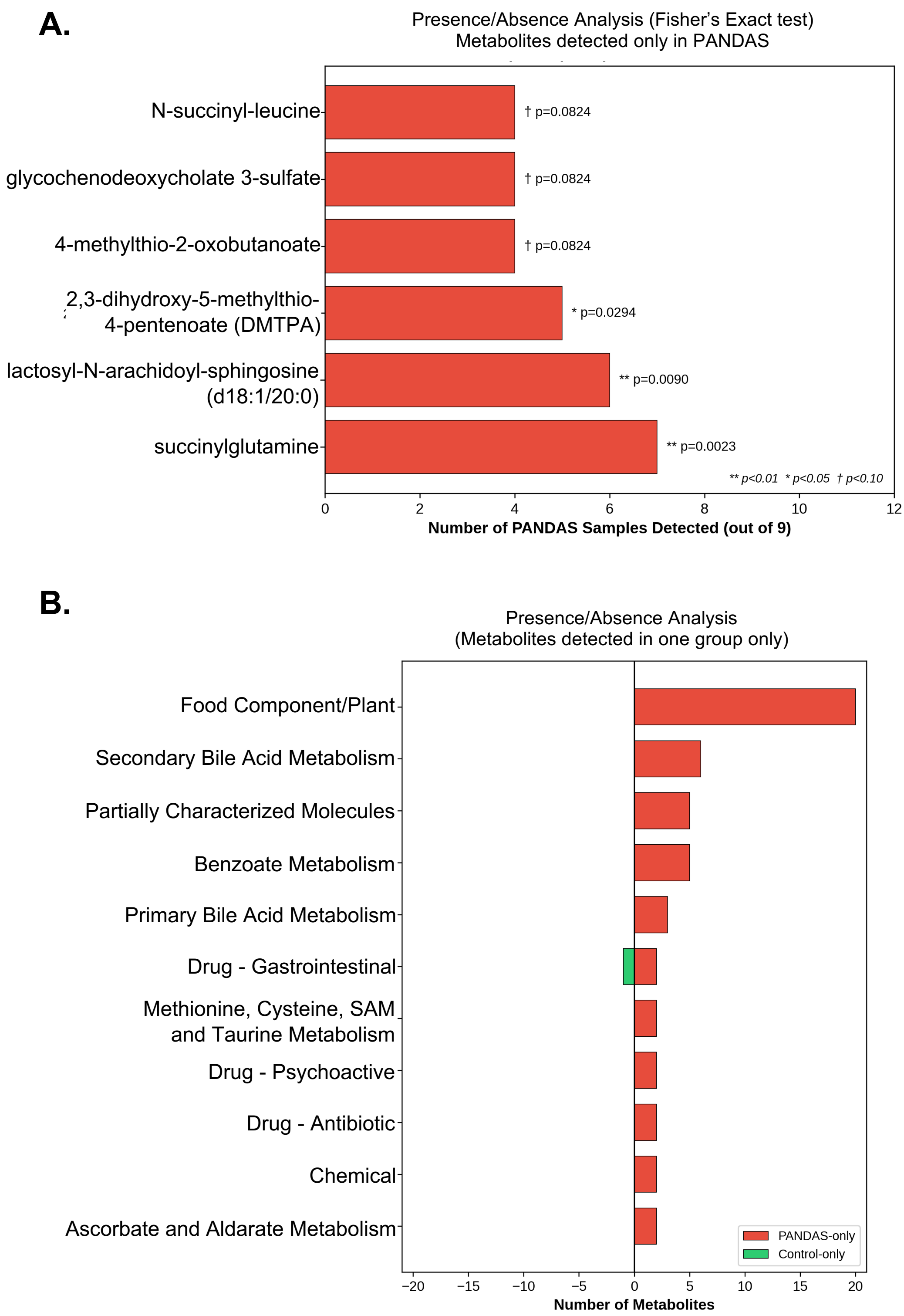

Supplemental Fig. S4: Presence/absence analysis of fecal metabolites. (A) Distribution of metabolites detected exclusively in PANDAS or control samples, categorized by metabolic pathway. **(B)** Metabolites uniquely detected in PANDAS patients (Fisher's exact test), including methionine pathway intermediates (4-methylthio-2-oxobutanoate, DMTPA), bile acid conjugates (glycochenodeoxycholate 3-sulfate), and lactosylceramides, supporting altered sulfur amino acid and sphingolipid metabolism in PANDAS.

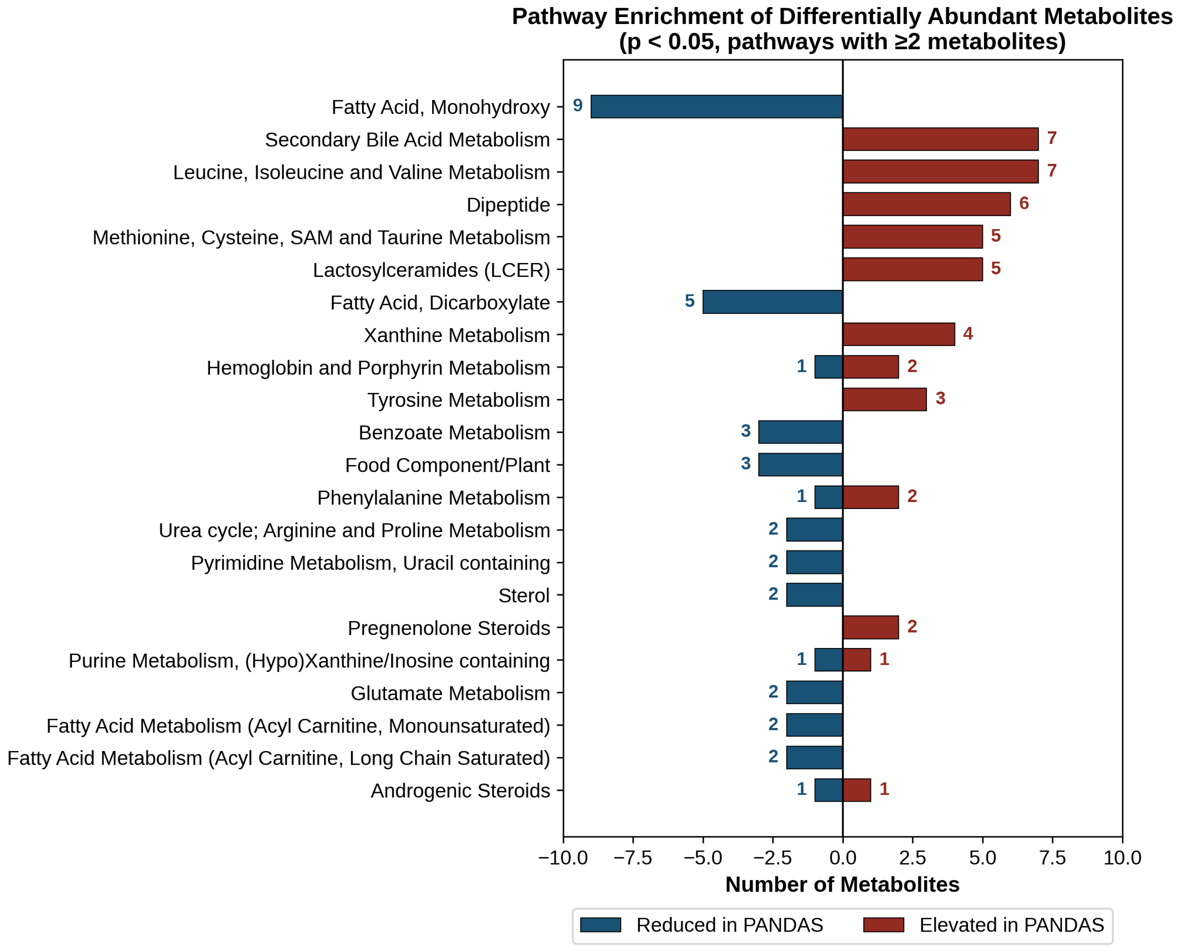

Supplemental Fig. S5: Pathway enrichment analysis of differentially abundant metabolites. Metabolic pathways significantly enriched among altered metabolites in PANDAS patients compared to controls. Key enriched pathways include branched-chain amino acid metabolism, methionine/sulfur amino acid metabolism, sphingolipid metabolism, and bile acid pathways, reflecting the functional consequences of gut microbiome dysbiosis on host metabolism.
